# Screening of M6a methylated genes associated with preovulatory follicle development in Bashang long-tailed chicken

**DOI:** 10.1016/j.psj.2026.106892

**Published:** 2026-04-01

**Authors:** Xiaoyan Duan, Jiale Xie, Shaoru Yin, Liyong Zhang, Yu Liu

**Affiliations:** Hebei North University, No.11 South Zuanshi Road, Zhangjiakou, Hebei 075000, China

**Keywords:** m6A methylation, Preovulatory follicle, MeRIP-seq, Bashang Long-tailed chicken

## Abstract

N6-methyladenosine (m6A) methylation, a pivotal post-transcriptional modification, regulates RNA metabolism and biological processes, including reproduction. However, its role in poultry follicular development remains unclear. This study integrated MeRIP-seq and RNA-seq analyses to profile m6A modifications and gene expression in preovulatory follicles of Bashang Long-tailed chickens. We identified 14,024 m6A peaks in small yellow follicles(SYF) and 14,063 in small white follicles(SWF) . A total of 651 differential m6A peaks (DMPs), with 562 upregulated and 89 downregulated in SWF compared to SYF were identified. Transcriptome analysis identified 3,250 differentially expressed genes (DEGs), including 319 upregulated and 2,931 downregulated in SWF. Integrated multi-omics analysis identified 95 genes with concurrent m6A and expression changes, of which *MAP1LC3C* were identified as target of post-transcriptional m6A regulation. We then constructed the regulation network of *MAP1LC3C* gene in pre-ovulatory follicle development of chickens. These findings provide insights into epigenetic mechanisms underlying avian reproduction, offering potential targets for improving egg production in native breeds of China.

## Introduction

N6-methyladenosine methylation is the most prevalent internal post-transcriptional modification in eukaryotic mRNA, occurring at the sixth nitrogen atom of adenine (Zhang and Xia, 2024). This dynamic and reversible process is regulated by methyltransferases, demethylases, and binding proteins. Recent studies have demonstrated that m6A modification plays a central role in RNA splicing, transport, stability, translation, and degradation, participating extensively in critical biological processes such as mammalian embryonic development, stem cell renewal and differentiation, gametogenesis, immune response, and tumor progression (Li et al., 2024; Wang et al., 2025). In the female reproductive system, m6A modification is particularly important. Mu et al. (2021) found that oocyte-specific knockout of *METTL3* leads to infertility in female mice, accompanied by impaired follicular development, abnormal ovulation, and premature ovarian failure, indicating that m6A is essential for normal oocyte meiosis and follicular health. Another study of Shi et al. (2017) revealed that m6A-recognizing proteins *YTHDF2* and *YTHDF3* coordinate mRNA stability of key genes through distinct degradation pathways during somatic cell reprogramming, thereby influencing cell fate transitions. This highlights the pivotal role of m6A-mediated post-transcriptional regulation in cellular state changes. In chickens, recent research has shown that m6A methylation regulates gonadal sex differentiation (Li et al., 2022) and ovarian maturation during sexual maturation (Jia et al., 2023), underscoring its significance in avian reproductive biology.

The Bashang Long-tailed chicken is a unique meat-and-egg dual-purpose local breed in Hebei Province, China, valued for its exceptional cold resistance, tolerance to extensive feeding, and strong adaptability, making it a precious avian genetic resource(Xie et al., 2017). However, compared to commercial high-yield egg-laying breeds, the egg-laying performance of Bashang Long-tailed chickens (approximately 160 eggs in 300 days) remains suboptimal, necessitating genetic analysis of its reproductive traits. Egg-laying performance is primarily determined by ovarian activity, with the developmental dynamics of preovulatory follicles being a core limiting factor (Du et al., 2020). Ovaries store numerous undifferentiated pre-ovulatory follicles, only a small fraction of which are recruited and selected to develop into ovulatory follicles. Thus, the developmental potential, recruitment efficiency, and conversion capacity of pre-ovulatory follicles directly determine egg production (Ni et al., 2023; Shang et al., 2020; Wang et al., 2024). While signaling pathways and hormones are known to regulate follicle development, the epigenomic mechanisms, particularly the role of m6A methylation in chicken follicular development, remain unclear.

This study used Bashang Long-tailed chickens at peak laying age (30 weeks) as a model to collect SWF and SYF. By integrating MeRIP-seq and RNA-seq, we aimed to: (1) map the m6A methylation profile of pre-ovulatory follicles in Bashang long-tailed chickens for the first time; (2) identify m6A-methylated genes that differentiate SWF and SYF developmental stages; and (3) integrate multi-omics data to screen key genes and signaling pathways regulated by m6A modifications. The findings provide new insights into the regulatory role of m6A methylation in avian follicular development and establish a theoretical foundation for discovering molecular targets to enhance egg production performance in native chicken breeds from an epigenetic perspective.

## Materials and methods

### Animal experiments and sample collection

Bashang Long-tailed chickens (n = 5) were reared in the animal laboratory of Hebei North University’s South Campus under standard feeding and management protocols. At 30 weeks of age (peak laying period with an egg production rate exceeding 80%), three chickens were randomly selected and euthanized to collect follicles of varying sizes. Follicles were classified by diameter, with SWF (n = 3) and SYF (n = 3) extracted (Three biological replicates, each representing a pool of follicles from an individual hen, were used for each follicle type). Samples were immediately immersed in liquid nitrogen for sequencing library preparation and subsequent experiments. All procedures were approved by the Experimental Animal Welfare Ethics Committee of Hebei North University.

### Library construction and sequencing

Total RNA was isolated and purified using TRIzol reagent (Invitrogen, Carlsbad, CA, USA). Poly(A) RNA was purified from 50 μg total RNA using Dynabeads Oligo(dT)25-61005 (Thermo Fisher, CA, USA) through two rounds of purification. The poly(A) RNA was fragmented into small pieces using the Magnesium RNA Fragmentation Module (NEB, cat. e6150, USA) at 86°C for 7 min. The cleaved RNA fragments were incubated for 2 h at 4°C with m6A-specific antibody (No. 202003, Synaptic Systems, Germany) in IP buffer (50 mM Tris-HCl, 750 mM NaCl, and 0.5% Igepal CA-630). The immunoprecipitated RNA was reverse-transcribed to cDNA using SuperScript II Reverse Transcriptase (Invitrogen, cat. 1896649, USA), followed by synthesis of U-labeled second-stranded DNAs with E. coli DNA polymerase I (NEB, cat. m0209, USA), RNase H (NEB, cat. m0297, USA), and dUTP Solution (Thermo Fisher, cat. R0133, USA). An A-base was added to the blunt ends for adapter ligation. Dual-index adapters were ligated to the fragments, and size selection was performed with AMPureXP beads. After treatment with heat-labile UDG enzyme (NEB, cat. m0280, USA), the ligated products were amplified by PCR under the following conditions: initial denaturation at 95°C for 3 min; 8 cycles of denaturation at 98°C for 15 s, annealing at 60°C for 15 s, and extension at 72°C for 30 s; and final extension at 72°C for 5 min. Paired-end sequencing (PE150) was performed on an Illumina NovaSeq 6000 platform (LC-Bio Technology Co., Ltd., Hangzhou, China) according to the manufacturer’s protocol.

The mapped reads from each sample were assembled using StringTie (version 2.1.2) with default parameters. StringTie and ballgown were used to estimate expression levels of all transcripts and calculate FPKM (fragments per kilobase per million mapped reads) values for mRNAs. Differential expression analysis was performed with DESeq2 software between groups, with genes exhibiting a p-value < 0.05 and absolute fold change ≥ 2 considered DEGs. For visualization and comparative purposes, FPKM (fragments per kilobase per million mapped reads) values were also calculated. DEGs were subjected to enrichment analysis of GO functions and KEGG pathways.

### m6A analysis

Reads from all samples were aligned to the Gallus gallus reference genome using HISAT2 (version 2.2.1). The R package ExomePeak was used to predict m6A sites and differential methylation sites with parameters: p cutoff = 1, p_adj_cutoff = NULL, log2FC_cutoff = 0, and parallel = 4. Peaks were annotated using ANNOVAR. Motif prediction was performed with HOMER (version 4.1) using parameters: -size 200, -len 5,8,10.

Integration of RNA-seq and MeRIP-seq data identified genes with both m6A methylation and expression changes. Genes with a fold change > 2 and p-value < 0.05 were considered to show significant transcriptional and methylation differences between groups.

## Results

**Fig. 1:**
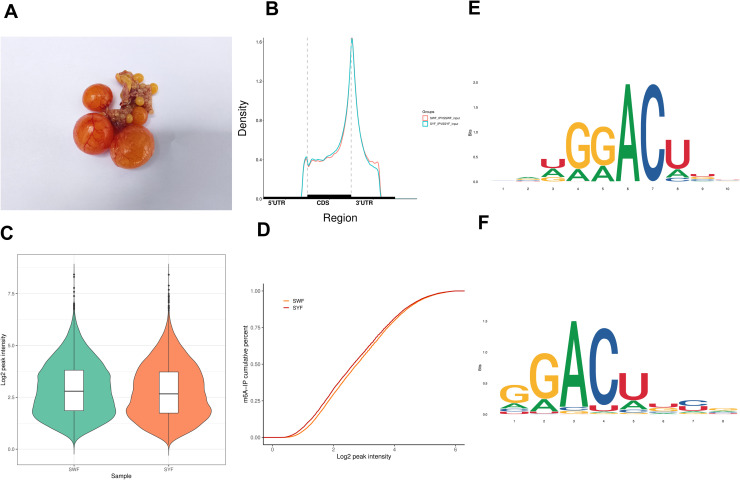
Overview of the m6A methylation profile in Bashang Long-tailed chicken preovulatory follicles. (A) The follicles of Bashang Long-tailed chicken. (B) Distribution curves of m6A modifications across different gene regions (5′UTR, CDS, 3′UTR) for SWF and SYF. (C) Violin plots comparing the distribution of Log2 peak intensity between SWF and SYF groups. (D) Cumulative percentage curves of m6A-IP signal relative to Log2 peak intensity for SWF and SYF groups. (E) Enriched RNA motifs identified from the m6A peaks. The logo uses colored letters (G, A, C, U) to represent nucleotide conservation and frequency at each position, with adenine (A) being highly prominent in both motifs shown.

In this study, we conducted transcriptomic analysis on SWF and SYF of Bashang long-tailed chickens (Fig. 1A) . The raw sequence data reported in this paper have been deposited in the Genome Sequence Archive, China National Center for Bioinformation / Beijing Institute of Genomics, Chinese Academy of Sciences (GSA: CRA035061) that are publicly accessible. MeRIP-seq generated 68,407,452 to 84,489,868 raw reads from IP or input samples of both follicle types. After quality control, over 67,822,874 high-quality reads were obtained per sample. Alignment to the reference genome identified 14,024 m6A peaks in SYF and 14,063 peaks in SWF.

Analysis of peak distribution across gene functional elements revealed that m6A methylation was predominantly enriched in CDS region of mRNA (37.2%) and near stop codons (28.4%) (Fig. 1B). Cumulative distribution differences in m6A peak intensity between SWF and SYF groups (Fig. 1C and D) indicated distinct m6A profiles, with SYF showing a higher concentration of RNA molecules with weak m6A modifications (Log2 peak intensity < 2).

Motif analysis of sequences surrounding m6A peaks identified the most significantly enriched motifs: NRWGGACTBB in SYF and GGACTKYG in SWF, both containing the classic RRACH sequence (Fig. 1E and F).

**Fig. 2:**
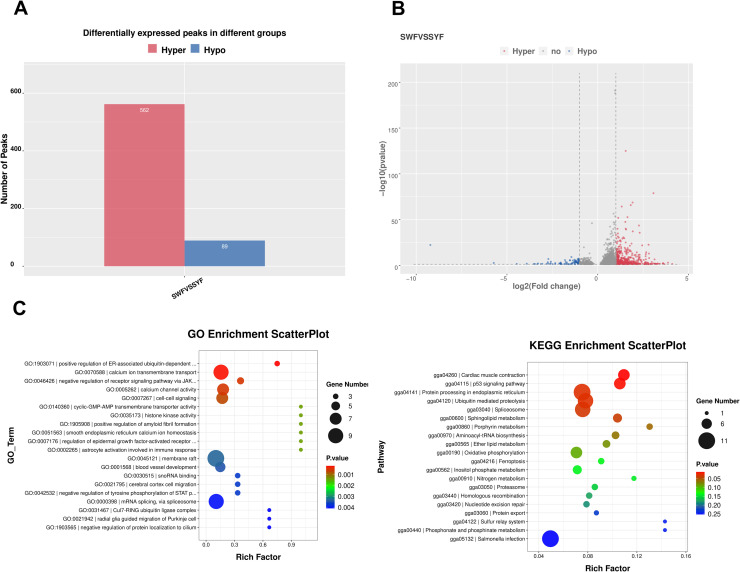
Identification and functional enrichment analysis of DMPs. (A) Number of hypermethylated (Hyper, 562) and hypomethylated (Hypo, 89) m6A peaks between SWF and SYF groups. (B) Volcano plot displaying the distribution of all m6A peaks. The x-axis represents the log2 fold change, and the y-axis shows the -log10 adjusted P-value. Data points are colored and labeled as hypermethylated (Hyper, red), hypomethylated (Hypo, blue), or non-significant (no, gray). (C) Scatter plots of functional enrichment analysis for genes associated with DMPs. Point size corresponds to the number of genes in each term, and point color indicates the enrichment significance (-log10 P-value). The left panel shows significantly enriched Gene Ontology (GO) terms, and the right panel shows enriched KEGG pathways. The Rich Factor on the x-axis represents the ratio of differentially methylated genes to all genes annotated in a given term.

Differential methylation peak analysis revealed 651 DMPs between SYF and SWF groups (Supplementary Table S1). The total length of DMPs was 3,583,532 bp, with an average length of 2,427.87 bp. Among these, SWF had significantly higher methylation levels in 562 peaks compared to SYF, while 89 peaks were down-regulated (Fig. 2A and B).

Functional enrichment analysis of methylated-differential genes identified 372 significantly enriched GO terms. The top five terms were: positive regulation of ER-associated ubiquitin-dependent protein catabolic process (GO:1903071), calcium ion transmembrane transport (GO:0070588), negative regulation of receptor signaling pathway via JAK-STAT (GO:0046426), calcium channel activity (GO:0005262), and cell-cell signaling (GO:0007267) (Fig. 2C). KEGG analysis revealed six enriched pathways: cardiac muscle contraction, p53 signaling pathway, protein processing in the endoplasmic reticulum, ubiquitin-mediated proteolysis, and spliceosome (Fig. 2D).

We conducted further analysis of DEGs in pre-ovulatory follicles of Bashang long-tailed chickens. RNA-seq data revealed 23,060 genes were expressed in these follicles. Using the threshold of |log2fc|≥1 & p < 0.05, we identified 3,250 DEGs in SYF and SWF groups, including 2,315 protein-coding genes, 928 lncRNAs, 4 mitochondrial tRNAs, 1 miRNA, and 1 pseudogene. The results are detailed in the Supplementary Table S2.

**Fig. 3:**
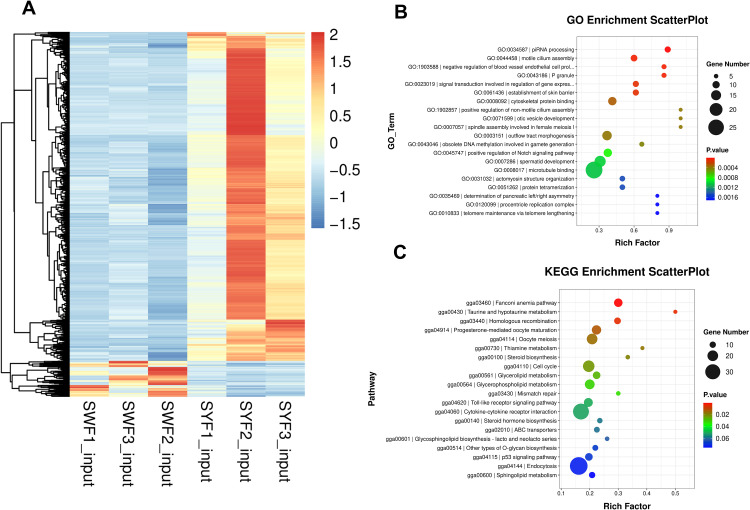
Gene expression clustering and functional enrichment analysis of DEGs. (A) Hierarchically clustered heatmap of DEGs between SWF and SYF groups. (B) Scatter plots of functional enrichment analysis for DEGs between SWF and SYF groups.

Among the 3,250 DEGs, 319 were upregulated and 2,931 downregulated in the SWF group, as shown in Fig. 3A. GO analysis revealed significant enrichment of these genes in 318 GO terms (Fig. 3B). Notably, several follicle development-related GO terms were significantly enriched, including oocyte maturation (GO: 0001556), positive regulation of follicle-stimulating hormone secretion (GO: 0046881), positive regulation of Notch signaling pathway (GO: 0045747), regulation of cell population proliferation (GO: 0042127), and obsolete DNA methylation involved in gamete generation (GO: 0043046). Pathway analysis revealed that the differential expression genes were significantly enriched in 11 signaling pathways, including progesterone-mediated oocyte maturation, oocyte meiosis, steroid biosynthesis, and cell cycle—pathways known to be associated with chicken follicular development. The results are shown in Fig. 3C.

**Fig. 4:**
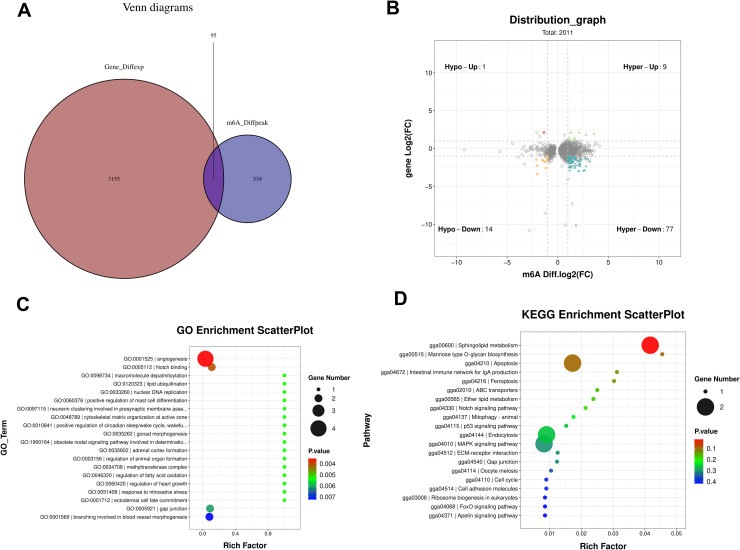
Integrated analysis of DEGs and m6A modifications, with functional enrichment. (A) Venn diagram between DEGs and DMPs. The intersection represents genes that are both differentially expressed and contain differential m6A modification. (B) Conjoint analysis m6A-seq and RNA-seq data (gray dots indicating genes with no significant differences; colored dots indicating genes with significant differences). (C) Scatter plots of GO analysis of genes with both m6A methylation and expression changes. (D) Scatter plots of KEGG analysis of genes with both m6A methylation and expression changes.

Integration of omics data identified 95 genes with both m6A methylation and expression changes. A Venn diagram (Fig. 4A) and four-quadrant plot (Fig. 4B) visualized these genes, revealing 78 genes with opposite trends in m6A modification and expression levels: one gene with down-regulated m6A peak and up-regulated expression, and 77 genes with up-regulated m6A peak and down-regulated expression. Functional enrichment analysis indicated that these genes were involved in follicular development pathways, including Notch signaling, MAPK signaling, and FoxO signaling (Fig. 4C and D).

**Fig. 5:**
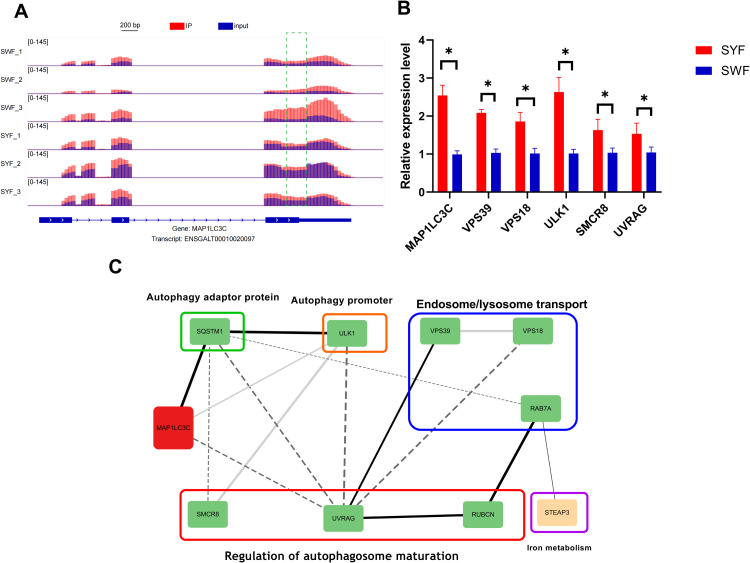
Integrated analysis of the *MAP1LC3C* m6A modification landscape and its associated autophagic regulatory network. (A) Visualization of m6A-seq peaks at the *MAP1LC3C* genomic locus. The top panel shows a genome browser view of the *MAP1LC3C* gene. Multiple tracks display the sequencing coverage (read density) for both MeRIP-seq IP (red) and input (blue) samples from biological replicates of the SWF and SYF groups. The bottom panel is a bar graph quantifying the normalized m6A enrichment (IP/Input signal ratio), clearly highlighting the differential peak (higher in SWF) at the 3′-end of the *MAP1LC3C* gene between SWF and SYF conditions. (B) Relative expression level of *MAP1LC3C* gene and its associated genes. (C) Protein-protein interaction network associated with genes harboring differential m6A modifications.

Notably, this study found that the *MAP1LC3C* gene, which plays a crucial role in both ferroptosis and autophagy pathways, showed significantly upregulated expression levels in the SYF group while exhibiting down-regulated m6A peaks. The visualization of m6A methylation modifications revealed that in the 3 '-end region of the *MAP1LC3C* gene, SWF group demonstrated significantly higher m⁶A enrichment signals (red) compared to SYF group, with high consistency across three replicates within the SWF group. However, the height, coverage, and position of SWF group's IP peaks did not match those of the SYF group. Furthermore, we identified altered expression levels in autophagy-related genes (*VPS39, VPS18, RAB7A, OPTN, SQSTM1, ULK1, SMCR8, UVRAG*, and *RUBCN*) and ferroptosis-related genes (*FTL, STEAP3*, and *SLC7A11*). Except for *SLC7A11*, which showed significant upregulation in the SWF group, all other genes exhibited significant upregulation in the SYF group. Real-time fluorescence quantification results for these genes corroborated the transcriptomic sequencing findings (Fig. 5B). Based on the functional roles of these genes in ferroptosis and autophagy, we constructed their functional networks, as illustrated in Fig. 5C.

## Discussion

The development of chicken follicles follows a strict hierarchical structure. Small white follicles, serving as undifferentiated follicle reservoirs, determine the pool size of available follicles for recruitment. Small yellow follicles, acting as a critical transitional phase for follicular maturation, directly influence ovulation potential through activation efficiency (Klein and Kues, 2025; Sun et al., 2022). This study, by integrating m6A methylation and transcriptome analyses, reveals epigenetic regulation and gene expression differences in prefollicular development at various stages of the Bashang Long-Tailed Chicken. The findings provide valuable insights for enhancing egg production performance in native chicken breeds. From a translational perspective, the identified m6A modification patterns and key genes like *MAP1LC3C* could serve as potential molecular markers for follicle selection. This opens up the possibility for future breeding strategies aimed at improving egg-laying performance by epigenetically selecting individuals with favorable m6A profiles associated with superior follicular development.

This study systematically analyzed the m6A methylation profiles of SWF and SYF in Bashang Long-tailed chickens using MeRIP-seq technology. The data revealed approximately 14,000 m6A peaks detected in both sample groups, covering over 5,000 genes, which were similar with other researches (Li et al., 2023; Fan et al., 2019), indicating widespread m6A modification in pre-ovulatory follicles. m6A peaks were predominantly enriched in CDS (37.2%) and near stop codons (28.4%), a distribution pattern highly consistent with classical m6A patterns observed in mammals (Zaccara et al., 2019). This suggests that m6A modification may influence gene expression in avian follicular development by regulating mRNA translation efficiency or stability. Notably, the SYF group exhibited significantly lower overall m6A levels (Log₂ peak intensity <2). Motif analysis revealed that the most significantly enriched motifs in both SYF and SWF contained the classic RRACH sequence, further validating the conservation of m6A modification and implying that follicular developmental stage transitions are accompanied by dynamic reprogramming of specific m6A modification patterns (Luo et al., 2022).

This study successfully identified 651 DMPs, with the SWF group showing significantly more up-regulated peaks (562) than the SYF group (89). This suggests that m6A modification may generally weaken during follicular development from SWF to SYF. Functional enrichment analysis revealed that differentially m6A-modified genes were significantly enriched in pathways such as endoplasmic reticulum protein processing, ubiquitin degradation, calcium signaling, and JAK-STAT signal regulation pathways known to participate in follicular granulosa cell proliferation, steroid hormone synthesis, and cell fate determination (Isola et al., 2024; Liu et al., 2023; Denef and Schüpbach, 2003). Crucially, integrated analysis showed that 95 genes exhibited both m6A modification and expression level changes, with 78 genes (approximately 82.1%) demonstrating opposite trends between m6A and expression levels. This negative correlation aligns with the classical model where m6A promotes mRNA degradation through reading proteins like *YTHDF2*, indicating that m6A may finely regulate the duration and intensity of follicular development-related gene expression at the post-transcriptional level (An and Duan, 2022; Zhang et al., 2023).

One of the most significant findings of this study lies in the *MAP1LC3C* gene. *MAP1LC3C* is a key protein in the autophagy process (Cho et al., 2021; Iriondo et al., 2022; Zhou et al., 2019;). Our data show that its mRNA expression levels significantly increased in the SYF group, while MeRIP-seq results revealed that the m6A enrichment signal in the 3 'end region of its transcript was markedly lower in the SYF group compared to the SWF group. The impact of m6A modification on gene expression is background-dependent, but extensive studies have shown that m6A modifications in the 3′ UTR region are typically recognized by "readers" such as *YTHDF2* and guide mRNA degradation (Zhang et al., 2020; Zhang and Tian, 2023). Therefore, the downregulation of m6A modifications in the 3′ UTR region of the *MAP1LC3C* gene likely reduces its degradation opportunities, thereby enhancing mRNA stability and ultimately leading to increased protein expression levels, which activates the autophagy flux. This discovery provides a new perspective for understanding the precise regulation of key functional genes during follicular screening.

In addition to *MAP1LC3C*, this study also found that multiple autophagy-related genes (such as *VPS39, ULK1, SQSTM1*, etc.) and ferroptosis-related genes (such as *FTL, STEAP3*) were consistently upregulated in the SYF group. Notably, autophagy and ferroptosis, as two important cellular processes, exhibit close interactions (Liu et al., 2020; Gao et al., 2022). For example, Jeong et al. (2019) found that the expression changes of *SQSTM1/p62*, a autophagy adapter protein, directly affect autophagy activity; while the upregulation of *FTL* may influence ferroptosis sensitivity by regulating intracellular iron homeostasis (Lei et al., 2023). The coordinated upregulation of these genes strongly suggests that during the dominance process of follicular development from SWF to SYF, autophagy and ferroptosis pathways may be jointly activated, forming a synergistic network. This network promotes granulosa cell health by clearing damaged organelles and maintaining redox balance, thereby supporting the survival and maturation of dominant follicles. The sole exception is *SLC7A11*, which is upregulated in the SWF group. *SLC7A11* is a known iron death-related factor which may indicate that in follicles approaching closure, cells attempt to suppress ferroptosis by upregulating *SLC7A11*, but ultimately succumb to the overall pro-death signaling.

Based on these findings, we propose a working model: During the critical stage of follicular development from SWF to SYF, m6A methylation undergoes global reprogramming. Specific m6A "writing" or "erasing" enzymes such as *METTL3, FTO*, may be activated, targeting mRNA of development-related genes to regulate their stability by altering modification levels. Genes such as *MAP1LC3C* with down-regulated m6A exhibit increased expression, thereby activating downstream pathways like autophagy and affecting granulosa cell functional states; conversely, m6A-upregulated genes show suppressed expression. Which suggesting that m6A may interact with different signaling pathways to form a complex regulatory network determining whether follicles proceed toward dominant maturation or atresia.

## Conclusion

In summary, this study has pioneered the mapping of m6A methylation profiles in pre-ovulatory follicles of Bashang Long-tailed chickens, revealing coordinated m6A modifications and gene expression patterns during follicular development. Notably, key genes including *MAP1LC3C* were identified as targets of post-transcriptional m6A regulation. These findings provide new evidence for elucidating epigenetic mechanisms underlying avian reproductive performance. These efforts will facilitate the discovery of molecular targets for enhancing avian egg production performance through epigenomic transcriptomic approaches.

## CRediT authorship contribution statement

**Xiaoyan Duan:** Writing – review & editing, Writing – original draft, Methodology, Formal analysis, Data curation, Conceptualization. **Jiale Xie:** Validation, Resources, Investigation. **Shaoru Yin:** Visualization, Software, Investigation. **Liyong Zhang:** Visualization, Resources, Project administration, Funding acquisition. **Yu Liu:** Writing – review & editing, Writing – original draft, Supervision, Funding acquisition, Conceptualization.

## Disclosures

The authors declare no conflict of interest.

## Appendix Supplementary materials

Image, application 1

Image, application 2

## Footnotes

Supplementary material associated with this article can be found, in the online version, at doi:10.1016/j.psj.2026.106892.
